# Bilateral spontaneous chylothorax after severe vomiting in children

**DOI:** 10.1016/j.rppede.2016.04.003

**Published:** 2016

**Authors:** Antonio Lucas Lima Rodrigues, Mariana Tresoldi das Neves Romaneli, Celso Dario Ramos, Andrea de Melo Alexandre Fraga, Ricardo Mendes Pereira, Simone Appenzeller, Roberto Marini, Antonia Teresinha Tresoldi

**Affiliations:** a Hospital de Clínicas da Universidade Estadual de Campinas (Unicamp), Campinas, SP, Brazil; b Faculdade de Ciências Médicas da Universidade Estadual de Campinas (Unicamp), Campinas, SP, Brazil

**Keywords:** Chylothorax, Vomiting, Thoracic duct, Scintigraphy, Child

## Abstract

**Objective:**

To report the case of a child with bilateral chylothorax due to infrequent etiology: thoracic duct injury after severe vomiting.

**Case description:**

Girl, 7 years old, with chronic facial swelling started after hyperemesis. During examination, she also presented with bilateral pleural effusion, with chylous fluid obtained during thoracentesis. After extensive clinical, laboratory, and radiological investigation of the chylothorax etiology, it was found to be secondary to thoracic duct injury by the increased intrathoracic pressure caused by the initial manifestation of vomiting, supported by lymphoscintigraphy findings.

**Comments:**

Except for the neonatal period, chylothorax is an infrequent finding of pleural effusion in children. There are various causes, including trauma, malignancy, infection, and inflammatory diseases; however, the etiology described in this study is poorly reported in the literature.

## Resumo

**Objetivo:**

Relatar o caso de uma criança com quilotórax bilateral devido a etiologia pouco frequente: lesão do ducto torácico após quadro de vômitos excessivos.

**Descrição do caso:**

Menina, sete anos, apresentava edema facial crônico iniciado após quadro de hiperemese. À avaliação, também apresentava derrame pleural bilateral, com líquido quiloso obtido na toracocentese. Após extensa investigação clínica, laboratorial e radiológica da etiologia do quilotórax, foi definido ser secundário a lesão do ducto torácico por aumento da pressão intratorácica pela manifestação inicial de vômitos, corroborado por achados de linfocintilografia.

**Comentários:**

À exceção do período neonatal, o quilotórax é achado infrequente de efusão pleural em crianças. As causas são diversas, incluindo trauma, neoplasia, infecção e doenças inflamatórias; contudo, etiologia como a aqui descrita é pouco relatada na literatura.

## Introduction

Chylothorax is defined as lymph accumulation in the pleural space, caused by injury to the thoracic duct and is a rare cause of pleural effusion in children.^1^^,^ 2 It can lead to significant respiratory morbidity and has an extensive list of causes, with great diagnostic difficulty.^1^^,^ 2 This study aims to report the case of a child with spontaneous bilateral chylothorax.

## Case description

Seven-year-old white female patient, referred due to suspected diagnosis of systemic lupus erythematosus. She had a five-month history of sudden-onset vomiting and self-limited abdominal bloating after ingestion of large amounts of chocolate; subsequently, she started to show insidious and permanent chronic swelling of face. Three months after symptom onset and extensive evaluation of allergies, she was submitted to a chest and abdomen computed tomography, which showed abdominal lymphadenomegaly and bilateral pleural effusion. Chest drainage was performed in another service and the presence of milky pleural fluid was reported. She also underwent laboratory evaluation at the original service and most results were within normal values (including whole blood count, renal function, C3, C4, rheumatoid factor, anti-Sm, anti-Ro, anti-La, anti-ds-DNA), except for a positive antinuclear antibody, at a titration of 1:640, nuclear speckled pattern.

**Figure 1:**
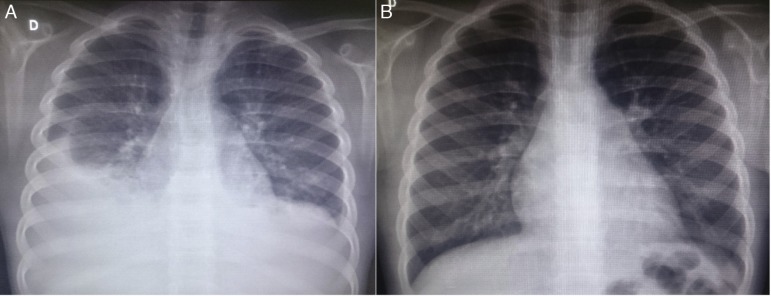
Chest radiography images of the patient in the posteroanterior view; A-at patient admission, costophrenic sinus obliteration is observed bilaterally, with pleuropulmonary opacity to the right; B-six months after discharge, during an outpatient consultation, the radiography shows no alterations.

At the first outpatient visit in our service, the patient underwent a new chest radiography (Fig. 1A), which showed recurrence of bilateral pleural effusion. A thoracentesis was performed on the right, of which milky white fluid showed the presence of 1.120mm^3^ of leukocytes (96% lymphocytes, 3% neutrophils, 1% plasma cells); 710mm^3^ of red blood cells; 3.7g/dL of protein; 87mg/dL of Glucose; 2.855mg/dL of triglycerides. The child was hospitalized, kept in fasting and started parenteral nutrition therapy. After 21 days without reduction in the chylothorax volume, bilateral thoracic drainage was performed and 450mL of chylous secretion was removed from the right and 300mL from the left side. The drains were maintained in water seal, with a marked reduction in eyelid edema. Three days after the draining she was started on a low-fat diet. The drains were removed after 25 days.

**Figure 2:**
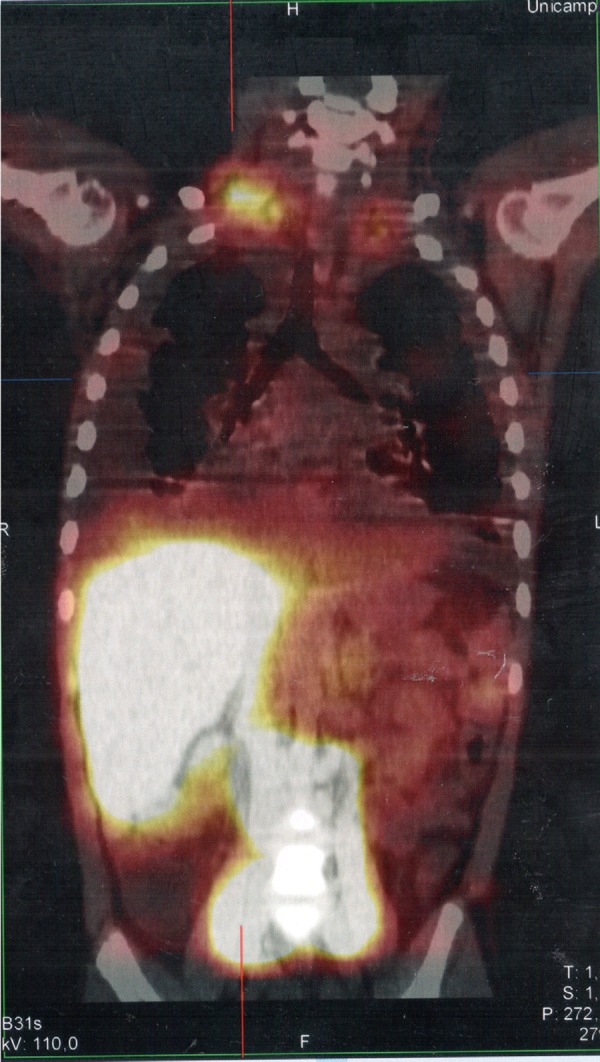
SPECT/CT image of the cervicothoracic and abdominal regions obtained from the lymphoscintigraphy assessment with intradermal administration of dextran-99mTc on the dorsum of the feet; the images obtained 18hours after start of the examination show focal area of radiotracer retention/leakage in the lymph ducts located in the topography of the thoracic introit, bilaterally, more accentuated to the right.

Serum levels of vascular-endothelial growth factor-D (VEGF-D), a marker which, at high levels, is useful for the diagnosis of lymphangioleiomyomatosis, was requested and the result of 125pg/mL, a little above the reference value (31-86pg/mL), ruled out that possibility. She underwent a lymphoscintigraphy (Fig. 2) with intradermal administration of the radiopharmaceutical in the instep and subsequent uptake of the radiotracer images that showed its extravasation in the topography of the thoracic introit bilaterally, compatible with thoracic duct lesions, secondary to increased intracavitary pressure caused by vomiting that occurred at the beginning of the clinical picture. The patient was discharged after 53 days of hospital stay, with outpatient follow-up, during which she was allowed to resume a normal diet without lipid restrictions. She remains asymptomatic at the follow-up (control chest X-ray in Fig. 1B), without alterations in the laboratory tests, including evaluation by a rheumatologist.

## Discussion

The clinical picture of a patient with chylothorax is insidious and develops as fluid accumulates in the pleural cavity; asymptomatic at first, it then progresses with cough and dyspnea^1^^,^ 2; fever and pleuritic pain are rare findings.^2^^,^ 3 On physical examination, there is unilateral or bilateral dullness and decreased breath sounds.^3^ Complications of a chylothorax with chronic evolution include protein-calorie malnutrition, immune deficiency by lymphocyte and immunoglobulin depletion^1^^,^ 4 and electrolyte disorders.^5^

The diagnosis, based on clinical suspicion in patients with suggestive clinical picture and compatible radiological findings (pleural effusion in the plain chest X-ray or ultrasound), is attained through the thoracocentesis.^1^^,^ 3 The fluid removed from the pleural cavity is white, odorless, milky white,^2^^,^ 3 but can be as serosanguinous.^3^ Laboratory evaluation showed triglyceride levels in the sample above of 110mg/dL and cholesterol ratio of pleural fluid over serum<1.0.^1^^,^ 3 Usually, cellularity is predominantly comprised of lymphocytes (>50%), with a protein content between 2 and 3mg/dL^5^ and low levels of lactate dehydrogenase.^3^^,^ 5 If the diagnostic doubt persists, the preferred method is chylomicron analysis in the biological fluid, with a positive result.^1^^,^ 3

After diagnostic confirmation, one can use other tests to help in the investigation, such as computed tomography and/or magnetic resonance imaging, as well as more specific tests to evaluate the lymphatic system, such as lymphangiography and lymphoscintigraphy.^1^^,^ 2 According to the location of the thoracic duct rupture, while also considering the anatomical variations, unilateral collection (most commonly on the right) can be detected or, more rarely, bilateral (in one-sixth of cases).^5^

The causes of chylothorax in children are diverse, varying according to age and the thoracic duct lesion mechanism. A review published in 2014 reports more than 35 possible etiologies.^1^ Among these are congenital malformations of the lymphatic system, such as pulmonary lymphangioma, lymphangiectasia and thoracic duct atresia^1^^,^ 2 ^,^ 6; chylothorax associated with genetic syndromes, such as Down, Noonan and Turner syndrome,^6^ among others^1^^,^ 6; after head and neck and thoracic surgical procedures (in up to 6% of cardiac surgeries)^1^^,^ 7; after other iatrogenic events in the neonatal period, such as birth trauma and superior vena cava thrombosis due to central venous catheterization^5^^,^ 6; chylothorax after closed thoracic trauma^1^; and chylothorax associated with cancer, such as neurogenic neoplasia, teratomas, sarcomas and especially lymphomas, in which the lymph accumulation in the pleural space may be the initial manifestation,^1^^,^ 2 in addition to granulomatous infections such as tuberculosis.^1^ The patient in this case did not have findings that were consistent with congenital malformations, had not suffered trauma or surgery, whereas cancer and infections were ruled out. Other possible causes for the development of chylothorax are the rheumatological ones, the initial reason why our patient came to the service. Possible triggers that have been described are systemic lupus erythematosus,^8^ Behçet's disease,^9^ Henoch-Schönlein purpura^10^ and sarcoidosis.^11^ Likewise, the patient showed no clinical and laboratory criteria for these conditions before or during the follow-up, which were then ruled out.

Another condition ruled out in this case was lymphangioleiomyomatosis. It is a rare disease that can be associated with the tuberous sclerosis complex, is characterized as low-grade metastasizing neoplasm, which leads to insidious cystic changes in the lung parenchyma and also affects the lymph vessels and lymph nodes and leads to chylothorax.^12^ The VEGF-D measurement was used to rule out this diagnosis in this case, as the marker is present at high levels in most patients and it is considered reliable for the diagnosis and evaluation of therapeutic response in these cases.^12^^,^ 13

Therefore, after excluding all possibilities, the remaining cause was considered for the onset of chylothorax in a child: increased intrathoracic pressure caused by excessive coughing or vomiting.^1^ This condition is associated with diseases such as Boerhaave and Mallory-Weiss syndromes, pneumothorax and subcutaneous emphysema.^14^ However, the association of this condition with thoracic duct rupture is rarely described-there is one report of a nine-year-old girl with chylothorax after excessive vomiting^14^ and two adults (33 and 65) with the onset of lymphatic pleural effusion after coughing episodes.^15^^,^ 16 Considering the history of excessive vomiting and abrupt symptom onset, this cause could be inferred in the present case. However, contrary to reports in the literature, the lymphatic lesion in the patient described here occurred at the thoracic introit level and not near the diaphragm.^2^^,^ 15

The treatment of chylothorax after thoracentesis and eventual chest drainage is initially conservative, based on a fat-free diet with addition of medium-chain triglycerides, which are absorbed directly into the portal circulation.^1^^,^ 2 ^,^ 7 If the enteral diet fails or as a first optional choice, parenteral nutrition can be used.^2^^,^ 7 Adjuvant therapies, such as use of octreotide,^5^^,^ 7 still need more support in the literature for treatment in children.^1^ Due to other complications, such as hypogammaglobulinemia and the increased risk of thrombosis (due to the loss of antithrombin), some authors have recommended the use of intravenous immunoglobulin and anticoagulation, according to each case.^7^ The list of options for the surgical treatment includes thoracic duct ligation by thoracoscopy, pleurodesis and pleuroperitoneal shunts; however, these approaches, as a rule, are reserved for patients who do not respond to the initial medical treatment (no improvement after 2-4 weeks and maintenance of high drainage output).^1^^,^ 2 ^,^ 5 ^,^ 7 In addition to these specific therapies, eventual basal conditions such as malignancy or infection should be assessed, for better case resolution.^5^

Although it is a rare cause of pleural effusion in children (except for the neonatal period),^1^^,^ 6 chylothorax can result in significant morbidity in these patients. This case illustrates the difficulty of clarifying the diagnosis of the chylothorax cause and serves as a reminder that, despite a number of other diseases that are more often associated with this entity, thoracic duct injury due to increased intrathoracic pressure caused by significant coughing and/or vomiting should also be included in the differential diagnosis.
